# Analgesic, anti-inflammatory and anti-platelet activities of *Buddleja crispa*

**DOI:** 10.1186/s12906-016-1021-4

**Published:** 2016-02-25

**Authors:** Ishfaq A. Bukhari, Anwar H. Gilani, Sultan Ayoub Meo, Anjum Saeed

**Affiliations:** Department of Pharmacology, College of Medicine, King Saud University, Riyadh, Saudi Arabia; Natural Product Research Unit, Department of Biological and Biomedical Sciences, Aga Khan University Medical College, Karachi, Pakistan; Prince Abdullah Bin Khalid Celiac Disease Research Chair, King Saudi University, Riyadh, Saudi Arabia; Department of Physiology, College of Medicine, King Saud University, Riyadh, Saudi Arabia

**Keywords:** *B. crispa*, Analgesic, Anti-inflammatory, Anti-platelet, Rodents

## Abstract

**Background:**

*Buddleja crispa* Benth (Buddlejaceae) is a dense shrub; several species of genus Buddleja have been used in the management of various health conditions including pain and inflammation. The present study was aimed to investigate the analgesic, anti-inflammatory and anti-platelet properties of *B. crispa*.

**Methods:**

Male rats (220–270 gm,) and mice (25–30 gm) were randomly divided into different groups (*n* = 6). Various doses of plant extract of *B. crispa*, its fractions and pure compounds isolated from the plant were administered intraperitoneally (i.p). The analgesic, anti-inflammatory and anti-platelet activities were assessed using acetic acid and formalin-induced nociception in mice, carrageenan-induced rat paw edema and arachidonic acid-induced platelets aggregation tests.

**Results:**

The intraperitoneal administration of the methanolic extract (50 and 100 mg/kg), hexane fraction (10 and 25 mg/kg i.p) exhibited significant inhibition (*P* < 0.01) of the acetic acid-induced writhing in mice and attenuated formalin-induced reaction time of animals in second phase of the test. Pure compounds BdI-2, BdI-H3 and BH-3 isolated from *B. crispa* produced significant (*P* < 0.01) analgesic activity in acetic acid-induced and formalin tests. The crude extract of *B. crispa* (50–200 mg/kg i.p.) and its hexane fraction inhibited carrageenan-induced rat paw edema with maximum inhibition of 65 and 71 % respectively (*P* < 0.01). The analgesic and anti-inflammatory effect of the plant extract and isolated pure compounds were comparable to diclofenac sodium. *B. crispa* plant extract (0.5–2.5 mg/mL) produced significant anti-platelet effect (*P* < 0.01) with maximum inhibition of 78 % at 2.5 mg/ml.

**Conclusion:**

The findings from our present study suggest that *B. crispa* possesses analgesic, anti-inflammatory and anti-platelet properties. *B. crispa* could serve a potential novel source of compounds effective in pain and inflammatory conditions.

## Background

*Buddleja crispa* Benth (Buddlejaceae) is a dense shrub endemic in Indo-Pak. The genus Buddleja comprises about 100 species found in the warmer parts of Southern Asia, Africa and America. The genus has diverse medicinal uses and several of its species have been used in a variety of health conditions worldwide [1]. *B. asiata*, indigenous to China and India is known for its use as an abortifacient and for treatment of skin diseases. The aqueous extract of *B. globase* is used for stomach ulcers, wounds and burns in central and southern regions of Chile. *B. officinalis* has a long traditional use as an adjuvant treatment of inflammatory and neuronal diseases in Korea and China [2].

Various species of genus Buddleja have been reported for pharmacological activities. *B. crispa* has shown blood pressure lowering, spasmolytic [3], antioxidant and lipoxygenase inhibitory effects [4]. *B. scordioides* exhibited gastroprotective, anti-inflammatory and antioxidant effects [5]. Inhibition of lipopolysaccharide-induced proinflammatory responses by *B. officinalis* has also been reported [2]. *B. cordata* possesses neuroprotective properties [6]. The phytochemical studies on genus Buddleja have revealed the isolation of various natural products, including sterols, aryl esters, triterpenoid glycosides, phenylethanoids [7], flavonoids [8], phenolic fatty acid esters [9] and diterpenes [10]. Some new constituents, such as buddlejoside (iridoid galactoside), buddlejone (sesquiterpene), aryl esters along with known compounds, β-sitosterol and ursolic acid have been isolated from *B. crispa* [4].

Owing to the diverse medicinal uses of the genus Buddleja in inflammatory conditions and the phytochemical diversity of the genus which is rich in flavonoids and terpenoids, the present study was undertaken to evaluate the analgesic and anti-inflammatory potential of *B. crispa* extract and some of its pure compounds, BdI-H3, BdI-2 and BH-3.

## Methods

### Plant material

The plant material was collected from Baluchistan in March 2003 and identified as *B.crispa* Benth., by Taxonomist at the Department of Botany, University of Baluchistan, Pakistan. A voucher specimen (BBU-101) was deposited at the herbarium of the same Department.

### Extraction and fractionation

The plant material (40 kg) was cleaned, shade dried, coarsely ground and soaked in 70 % aqueous-methanol for 7 days with occasional shaking. It was first filtered through a muslin cloth and then through a filter paper. This procedure was repeated thrice and the filtrate was combined and evaporated on rotary evaporator under reduced pressure (−760 mmHg) at 35–40 °C to obtain a thick, semi-solid mass of dark brown color; i.e. the crude extract (Bc.Cr), yielding approximately 800 gm. Approximately 250 gm of the crude extract of *Buddleja crispa* (Bc.Cr) was dissolved in about 300 mL of distilled water for fractionation with hexane, chloroform and Ethyl acetate. Pure compounds, Bdl-2, Bdl-H3 and BH-3 were isolated from *B. crispa.*

### Animals

Male NMRI mice (25–30 gm) and Sprage-Dawley rats (220–270 gm) obtained from the animal house facility of The Aga Khan University, Karachi and animal house facility of College of Medicine, King Saud University Riyadh, Saudi Arabia respectively. Animals were housed in plastic cages (6 rats per cage) under standard condition with 12 h light: dark cycle with free access to food and water.

### Chemicals

Acetic acid, arachidonic acid, carrageenan, diclofenac sodium and indomethacin were purchased from the Sigma Chemical Co., (St. Louis Mo, USA). Formalin 37 % was obtained from Fluka Chemie, Switzerland respectively. All other chemicals used in experiments were of analytical grade.

### Experimental Methods

#### Acute toxicity tests

Male mice (20–25 gm) were injected intraperitoneally (i.p) different doses of the *B. crispa* extract (50, 100, 300, 500, 1000 and 2000 mg/kg; *n* = 6) for observation. The animals were observed for 1–2 h after administration of the extract for any acute signs of behavioral toxicity. The number of deaths counted at 48 h after treatment. Dose of the extracts producing mortality in the 50 % of the experimental animals (LD_50_) was determined by graphical method [11].

#### Writhing test

Male mice (20–25 gm) were used in this experiment. 30 min. after the administration of *B. crispa* (50 and 100 mg/kg i.p), mice were administered an i.p. injection of 0.7 % v/v acetic acid solution. The mice were placed individually in transparent cages and 5 min were allowed to elapse. The number of acid-induced writhes were counted for 20 min. For the purpose of scoring, a writhe was indicated by stretching of the abdomen and/or simultaneous stretching of at least one hind limb. Control animals were injected normal saline (10 ml/kg, i.p.), diclofenac (10 mg/kg, i.p.) was used as a standard drug.

#### Formalin test

This test was performed according to method described by Hunskaar and Hole (1987) [12] male mice (20–25 gm) were injected, 20 μl of 1 % formalin prepared in 0.9 % saline, subcutaneously into the dorsal hind paw and transferred immediately in transparent box for observation. The duration of reaction time (paw licking or biting) was determined between 0–5 min (first phase) and 15–30 min (second phase) after formalin injection. Animals were administered different doses of *B. crispa* plant extract (50 and 100 mg/kg i.p.) or diclofenac (10 mg/kg, i.p.). Control animals received the vehicle (normal saline; 0.1 ml/10 gm). The reaction time of the animals was compared to control group and expressed as percent inhibition.

#### Anti-inflammatory activity

The carrageenan induced hind paw edema test was conducted according to the method of Winter et al. (1962) [13]. Male Rats (180–250 gm) divided randomly into different groups (*n* = 5–8) and injected 0.05 ml of freshly prepared 1 % carrageenan subcutaneously into the plantar surface of the hind paw of rats. Different doses of plant extract or diclofenac sodium were injected i.p., 30 min before the administration of carrageenan. The control animals received same volume of the vehicle (Saline or distilled water 1 ml/kg). Rat paw edema was assessed by volume displacement method (plethysmometer (Ugo Basile 7150) before and after carrageenan injection at 1, 2, 3 and 4 h. Difference in the paw volume, determined before and after injection of the phlogistic agent indicated the severity of edema. The % inhibition of the inflammation was determined for each animal by comparison with controls and calculated by the formula [14], % I = 1- (dt/ dc) x 100

Where “dt” is the difference in paw volume in the drug treated group and “dc” the difference in paw volume in control group and “I” = inhibition.

#### Anti-platelet effect

The anti-platelet activity of *B. cripa* was studied according to the method as described by Saeed et al. (1995) [15]. Blood was taken by venepuncture from normal volunteers who had not taken any medication for 1 week. Blood samples were mixed with 3.8 % (w/v) sodium citrate solution (9:1) and centrifuged at 260 gm for 15 min at 20 °C to obtain platelet rich plasma (PRP). Platelet poor plasma (PPP) was prepared by centrifugation of the the remaining blood sample at 1200 gm for 10 min. The aggregation studies were carried out at 37 °C. The aggregation was monitored with dual channel Lumi Aggregometer using 450 μl samples of PRP. The PRP was pre-incubated with appropriate amount of the test sample for 1 min. before challenge with aggregating agent (arachidonic acid). Aggregation was induced by arachidonic acid (0.8 mM) which was expressed as percentage inhibition compared with control at 5 min after challenge. Test samples were solubalized in 0.9 % saline or 10 % DMSO and this concentration did not interfere with platelet aggregation.

#### Ethical approval

The study protocol (08-ECACU-BBS-13) was approved by the Ethics Committee for Animal Care and Use (ECACU) of the Aga Khan University, Karachi, Pakistan.

#### Statistical analysis

The results of the study are expressed as mean ± S.E.M and statistical significance between control and treated groups was evaluated by one-way analysis of variance (ANOVA) followed by Tukey–Kramer multiple comparison test. *P* < 0.05 was considered statistically significant.

## Results

### Acute toxicity test

The intra-peritoneal administration of various dose of the plant extracts did not cause any lethality up to 1000 mg/kg. LD_50_ value of *B. crispa* was 1830 mg/kg. Animals showed hypo-motility, drowsiness at the dose range of 500–2000 mg/kg.

### Writhing test

As summarized in Table 1, the intraperitoneal (i.p) administration of the methanolic extract of *B. crispa* (25–100 mg/kg) caused significant inhibition (*P* < 0.01; *P* < 0.001) of the nociception induced by acetic acid. The plant extract produced maximum protection of 64 % at the dose of 100 mg/kg. The results were comparable to standard drug diclofenac sodium that produced 59 % inhibition at 10 mg/kg i.p (Table 1).

**Table 1:** Effect of *B. crispa* extracts and some pure compounds isolated from the plant on acetic acid induced writhing in mice

Treatment	Dose (mg/kg)	Number of writhing	% Protection
Control	-	73 ± 7	-
*B. crispa*	50	33 ± 5**	54
	100	26 ± 3***	64
Hexane fraction	10	32 ± 2***	56
	25	21 ± 2***	71
Aqueous fraction	50	38 ± 3**	48
BdI-2	5	35 ± 3**	52
	10	18 ± 5***	75
BdI-H3	1	49 ± 5*	33
	5	24 ± 4***	67
BH-3	5	56 ± 6	23
	10	48 ± 4*	34
Diclofenac sodium	10	30 ± 6***	59
	20	13 ± 5***	82

Values represent e mean ± S.E.M of 6-20 observations

*P < 0.05

**P < 0.01

***P < 0.001, compared to control

Among the various fractions the hexane fraction of *B. crispa* was the most potent with maximum protection of 71 % obtained at 25 mg/ kg. Aqueous fraction of the plant produced 48 % protection at 50 mg/kg. The analgesic potency of hexane fraction of *B. crispa* was 4-fold greater than its parent crude extract. The investigation of pure compounds isolated from the this plant i.e. BdI-2 (steroidal galacotside), BdI-H3 and BH-3 (aryl esters) exhibited significant analgesic effect (*P* < 0.01) against acetic acid-induced pain (Table 1).

### Formalin test

In the formalin test the plant extract of *B. crispa* (50 and100 mg/kg i.p) caused significant (*P* < 0.001) inhibition in the second phases of formalin induced pain. Maximum inhibition of 61 % was obtained at 100 mg/kg i.p in the second phase of formalin test (Table 2). *B. crispa* plant extract was ineffective in the first phase of formalin test. Similarly diclofenac sodium (10 mg/kg i.p) suppressed the reaction time of the animals in the second phase of the test only (Table 2). As shown in Table 2, the pure compounds BdI-2 (steroidal galacotside), BdI-H3 and BH-3 (aryl esters) isolated from the plant caused significant inhibition (*P* < 0.01) of the reaction time of animals in the second phase of the test.

**Table 2:** Effect of *B. crispa* and some pure compounds isolated from the plant in formalin test in mice

Treatment	Dose (mg/kg) i.p.	Licking time (Sec)		% Inhibition	
		1st Phase	2nd Phase	1st Phase	2nd Phase
Control	-	50 ± 3	75 ± 5	-	-
*B. crispa*	50	55 ± 3	42 ± 5**	-	44
	100	51 ± 4	29 ± 5***	-	61
Hexane fraction	10	47 ± 3	39 ± 5**	6	48
	25	52 ± 5	32 ± 6***	-	57
Aqueous fraction	50	43 ± 3	51 ± 5**	14	32
BdI-2	10	47 ± 5	25 ± 4***	-	67
				-	
BdI-H3	1	49 ± 6	53 ± 3*	-	29
	5	55 ± 7	46 ± 7**		38
BH-3	10	42 ± 7	37 ± 5**	16	50
Diclofenac sodium	20	47 ± 5	27 ± 6***	-	64

Values represent mean ± S.E.M of 6–30 observations

*P < 0.05

**P < 0.01

***P < 0.001, compared to control

### Anti-inflammatory activity

The subplantar injection of carrageenan produced a localized edema that reached to its maximum at the 3rd hour after injection. As shown in Table 3, *B. crispa* (50–200 mg/kg i.p.) caused significant inhibition (*P* < 0.001) of the carrageen induced inflammation. The maximum anti-inflammatory effect of the plant extract was 65 % at 200 mg/kg i.p. (Table 3). The anti-inflammatory effect of plant extract was evident at 3rd hour after carrageenan injection. Similarly diclofenace sodium and indomethacin, used as standard anti-inflammatory drugs, at 20 mg/kg i.p. produced about 76 and 59 % inhibition of the carrageenan induced edema respectively (Table 3). As shown in Table 3, Among various fractions of *B. crispa*, only hexane fraction exhibited significant (*P* < 0.01) anti-inflammatory activity which was comparable to crude extract of the plant.

**Table 3:** Effect of *B. crispa* and its fractions on carrageenan induced paw edema in rats

Treatment	Dose (mg/kg) i.p	Initial paw volume (ml)	Paw volume at 3 hr	Increase in paw volume	% Inhibition
Control	-	0.96 ± 0.01	1.13 ± 0.05	0.170	-
*B. crispa*	50	0.94 ± 0.03	1.04 ± 0.03	0.10**	41
	100	0.96 ± 0.04	1.02 ± 0.03	0.06***	65
	200	0.93 ± 0.03	0.99 ± 0.05	0.06***	65
Hexane fraction	50	0.95 ± 0.01	1.03 ± 0.02	0.08**	53
	100	0.95 ± 0.03	1.0 ± 0.01	0.05***	71
Aqueous fraction	50	0.98 ± 0.02	1.09 ± 0.02	0.11	15
Diclofenac sodium	20	0.98 ± 0.04	1.02 ± 0.03	0.04***	76
Indomethacin	20	0.96 ± 0.01	1.03 ± 0.02	0.07**	59

Values represent mean ± S.E.M of 6–30 observations

*P* < 0.05

** *P* < 0.01

*** *P* < 0.001, compared to control

### Anti-platelet effect

**Fig. 1:**
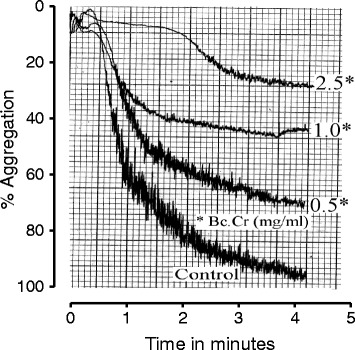
Tracing showing the inhibitory effect of crude extract of *B. crispa* (Bc.Cr) against arachidonic acid (0.8 mM) induced platelet aggregation. The platelet rich human plasma was incubated with different doses of the plant extracts or vehicle (control) for 1 min. before the addition of arachidonic acid

**Fig. 2:**
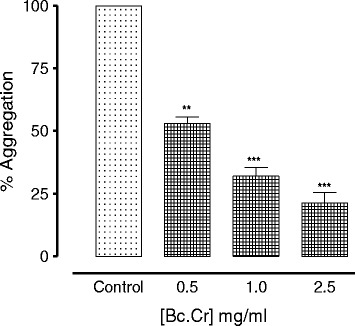
Inhibitory effect of crude extracts of *B. crispa* (Bc.Cr) on arachidonic acid induced platelets aggregation. Values represent the mean ± S.E.M of the % aggregation of the control maximum (*n* = 3-4), **P < 0.01 and ***P <0.001, compared to control

The inhibitory effect of *B. crispa* extract against platelet aggregation was assessed in the *in-vitro* experiments using arachidonic acid (AA) as the aggregating agent. pre-incubation of platelet rich plasma with crude extracts of *B. crispa* for 1 min. inhibited AA-induced platelet aggregation in dose dependent manner (Figs. 1 and 2). *B. crispa* extract (2.5 mg/ml) reduced platelet aggregation to 22 ± 3 (75 % inhibition) with IC_50_ value of 0.54 mg/ml, Fig. 2.

## Discussion

Various member of genus Buddleja including *B. globase* and *B. officinalis* are used in traditional medicine in the management of pain and inflammatory conditions [2]. The current study was planned to explore *B. crispa*, the least investigated plant of the genus, for possible analgesic and anti-inflammatory effects in animal model of pain and inflammation. The methanolic extract of the plant and its hexane fraction caused significant inhibition (*P* < 0.01) of the acetic acid induced writhes. Acetic acid increases the prostaglandins levels in the peritoneal fluid [16, 17] and commonly employed tool for screening analgesic agents. Diclofenac sodium, a standard non-steroidal anti-inflammatory, produced similar results, suggesting that the inhibitory effects of *B. crispa* plant extract against acetic acid induced writhing may have occurred through inhibition of prostaglandins action. Several medicinal plants used as analgesic such as *Moringa oleifera* [18], *Asparagus pubescens* [19] and *Melastoma malabathricum* [20] have been shown to decrease abdominal stretching /constriction induced by acetic acid.

The acetic acid induced writing test is effective but it is non-selective [21]. Acetic acid indirectly releases endogenous mediators which stimulate neurons that are sensitive to other drugs such as narcotic and other centrally acting drugs. The analgesic effect of *B. crispa* plant extract was therefore further investigated in the formalin test.

Formalin induced pain model is helpful to elucidate the mechanism of pain and analgesia [22]. Formalin test involves two distinct phases of analgesia, in the first phase (neurogenic phase) pain is produced due to direct stimulation of the sensory nerve fiber by formalin and in the second or late phase (inflammatory phase) the pain occurs due to release of inflammatory mediators such as histamine, prostaglandin and bradykinin [23]. It is well documented that centrally acting drugs such as narcotics inhibit both phases equally while peripherally acting drugs such as diclofenac inhibit the late phase [24–26]. In this study *B. crispa* produced marked analgesia in the second phase of the test similar to diclofenac, suggesting that peripheral mechanism is involved in analgesic effect of the plant extract. The predominant inhibitory effect of plant extract in the second phase of formalin test suggests that the anti-nociceptive effect of plant extract occurred via inhibition of prostaglandins synthesis [22]. The analgesic potency of hexane fraction of *B. crispa* was 4-fold greater than its parent crude extract and the aqueous fraction had weak inhibitory effect, showing the analgesic constituents concentrated in the hexane fraction. The investigation of pure compounds i.e. BdI-2 (steroidal galacotside), BdI-H3 and BH-3 (aryl esters) isolated from *B. crispa* exhibited profound analgesic activity in acetic acid induces pain and also in the late phase of formalin test, suggesting that these constituents contribute to the observed analgesic effect of the plant extract.

The marked inhibitory effect of plant extract of *B. crispa* and its fractions in the second phase of formalin test, similar to diclofenac, indicates its peripheral anti-inflammatory effect [23]. Therefore, the anti-inflammatory effect of *B. crispa* was assessed in carrageenan-induced inflammatory edema in the hind paw of rats. Carrageenan, a mucopolysacharide derived from Irish Sea moss Chondrus, is used to induce experimental arthritis. It is non-antigenic and does not produce any systemic effects [13]. The intraperitoneal administration of the methanolic extract of *B. crispa* caused significant (*P* < 0.001) inhibition of the late phase edema induced by the sub-plantar injection of carrageenan with no inhibitory effect in the initial phase of the test. Carrageenan produces acute inflammation believed to be biphasic; the early phase (1-2 h after carrageenan injection), in which the edema production is mediated by histamine and serotonin and the late phase (after 2nd h) the vascular permeability is maintained by bradykinin and prostaglandins [27]. These mediators contribute in the inflammatory response and induce pain [28]. It has been shown that the second phase of carrageenan-induced edema is sensitive to clinically used anti-inflammatory drugs and commonly employed to assess the anti-phlogistic effect of the natural products [29, 30]. In the present study the plant extract of *B. crispa* and its hexane fraction exhibited marked anti-inflammatory activity in the late phase of carrageenan induced edema test.

Similarly non-steroidal anti-inflammatory (NSAIDs) drugs diclofenac sodium and indomethacin produced significant (*P* < 0.001) anti-edematous effect which is consistent with the previous reports [31]. Several studies have revealed the inhibitory effects of plant extracts and NSAIDs in animal models of pain and inflammation [32–34]. It is known that diclofenac, and aspirin suppress inflammation and pain by inhibiting prostaglandin synthesis via inhibition of cyclooxygenase in arachidonic acid pathways [35]. The plant extract of *B. crispa* inhibited arachidonic acid induced platelet aggregation which further strengthening the NSAIDs like properties of of the plant extract.

## Conclusion

The plant extract and hexane fraction of *B. crispa* possesses analgesic and anti-inflammatory properties. *B. crispa* produced marked inhibition of the pain response in animals in chemical (acetic acid, formalin)- induced pain models. *B. crispa* also produced profound anti-phlogistic effect similar to standard drugs diclofenac sodium. The plant extract produced significant inhibition of arachidonic acid induced platelets aggregation. These finding suggest that *B. crispa* plant extract contains constituents with peripheral analgesic and anti-inflammatory properties. These finding validates the folkloric use of *B. crispa* and related species in the management of pain and inflammatory conditions. *B. crispa* could serve a natural resource for the discovery of novel compounds effective in pain and inflammatory conditions.
